# The Genotype of Early-Transmitting HIV gp120s Promotes α_4_β_7_ –Reactivity, Revealing α_4_β_7_^+^/CD4^+^ T cells As Key Targets in Mucosal Transmission

**DOI:** 10.1371/journal.ppat.1001301

**Published:** 2011-02-24

**Authors:** Fatima Nawaz, Claudia Cicala, Donald Van Ryk, Katharine E. Block, Katija Jelicic, Jonathan P. McNally, Olajumoke Ogundare, Massimiliano Pascuccio, Nikita Patel, Danlan Wei, Anthony S. Fauci, James Arthos

**Affiliations:** 1 Laboratory of Immunoregulation, National Institute of Allergy and Infectious Diseases, National Institutes of Health, Bethesda, Maryland, United States of America; 2 New York University School of Medicine Sackler Institute of Graduate Biomedical Sciences, New York, New York, United States of America; University of Zurich, Switzerland

## Abstract

Mucosal transmission of HIV is inefficient. The virus must breach physical barriers before it infects mucosal CD4^+^ T cells. Low-level viral replication occurs initially in mucosal CD4^+^ T cells, but within days high-level replication occurs in Peyer's patches, the gut lamina propria and mesenteric lymph nodes. Understanding the early events in HIV transmission may provide valuable information relevant to the development of an HIV vaccine. The viral quasispecies in a donor contracts through a genetic bottleneck in the recipient, such that, in low-risk settings, infection is frequently established by a single founder virus. Early-transmitting viruses in subtypes A and C mucosal transmission tend to encode gp120s with reduced numbers of N-linked glycosylation sites at specific positions throughout the V1-V4 domains, relative to typical chronically replicating isolates in the donor quasispecies. The transmission advantage gained by the absence of these N-linked glycosylation sites is unknown. Using primary α_4_β_7_^+^/CD4^+^ T cells and a flow-cytometry based steady-state binding assay we show that the removal of transmission-associated N-linked glycosylation sites results in large increases in the specific reactivity of gp120 for integrin- α_4_β_7_. High-affinity for integrin α_4_β_7_, although not found in many gp120s, was observed in early-transmitting gp120s that we analyzed. Increased α_4_β_7_ affinity is mediated by sequences encoded in gp120 V1/V2. α_4_β_7_-reactivity was also influenced by N-linked glycosylation sites located in C3/V4. These results suggest that the genetic bottleneck that occurs after transmission may frequently involve a relative requirement for the productive infection of α_4_β_7_^+^/CD4^+^ T cells. Early-transmitting gp120s were further distinguished by their dependence on avidity-effects to interact with CD4, suggesting that these gp120s bear unusual structural features not present in many well-characterized gp120s derived from chronically replicating viruses. Understanding the structural features that characterize early-transmitting gp120s may aid in the design of an effective gp120-based subunit vaccine.

## Author Summary

In the first days following sexual transmission, HIV replication occurs initially at relatively low levels in mucosal tissues because of a paucity of CD4^+^ T cell targets for the virus to infect. After a period of days, virus accesses specific gut tissues that are enriched in activated CD4^+^ T cells, where near-exponential replication ensues. The period of time before HIV accesses gut tissues represents a window of opportunity where a microbicide, pre-exposure and/or post-exposure antiretroviral prophylaxis or a vaccine-induced immune response could block infection. We previously reported that the HIV envelope protein gp120 binds to integrin α_4_β_7_ on the surface of CD4^+^ T cells. α_4_β_7_ mediates the homing of CD4^+^ T cells into the gut tissues where HIV can replicate exponentially. Here we report that the genotypic features that distinguish viruses isolated within the first month after infection, termed early-transmitting isolates, promote increased steady-state reactivity with α_4_β_7_. This property likely provides these viruses with enhanced transmission-fitness. These results suggest that the infection of α_4_β_7_^+^/CD4^+^ T cells can play an important role early in HIV transmission. These findings have potentially important implications in the design of interventions to block the mucosal transmission of HIV.

## Introduction

Despite widely available prevention modalities against HIV transmission, 2.6 million individuals are newly infected with HIV every year. Thus, there exists an urgent need for an effective HIV vaccine. A number of studies that have focused on the earliest events in HIV transmission raise the possibility that new strategies for an effective vaccine immunogen can be developed.

HIV transmission following mucosal exposure is inefficient[1]. The virus must first breach physical barriers in the mucosa, and then infect suitable target cells. In one study of heterosexual couples discordant for HIV infection, the frequency of transmissions per coital act averaged ∼0.01 [2]. One can therefore infer that, following deposition on the mucosal surface of the genital tract, HIV very frequently fails to establish infection. Both human and an SIV/macaque model studies indicate that during the first days of infection, termed the “eclipse phase”, low levels of viral replication occur, primarily in suboptimally activated memory CD4^+^ T cells in the genital mucosa[3], [4], [5], [6], [7], [8]. Although these cells are metabolically active, they do not express classical activation markers[4], [7]. Subsequently, HIV-1 infects fully activated memory CD4^+^ T cells. These events represent a critical point in transmission because they lead to high-level replication and the migration of virus into draining lymphoid tissue and ultimately gut-associated lymphoid tissue (GALT) where activated CD4^+^ T cells are plentiful, viral replication amplifies and the high level viremia that is associated with acute infection is established[9]. The best opportunity to prevent or abort establishment of HIV infection is likely during the eclipse phase following transmission, before HIV-1 migrates into the GALT.

A striking feature of sexual transmission of HIV is the extreme restriction in the genetic diversity of the viral quasispecies shortly after infection. The genetically diverse viral swarm replicating in an infected transmitting partner constricts through a genetic bottleneck in the course of sexual transmission such that transmission is usually the result of a single infectious event; the productive infection that follows, in most instances, reflects an expansion from a single founder virus [10], [11], [12], [13], [14], [15]. Importantly, this restriction occurs in the recipient, rather than in the transmitting partner. The early progeny of the transmitted founder virus show relative uniformity until adaptive immune responses drive the founder to diversify into a quasispecies. An instructive exception to this pattern occurs in recipients harboring certain sexually transmitted diseases (STDs). Inflammation in the genital mucosa, mediated by STDs, can promote transmission of multiple founder viruses due predominantly to the ready availability of activated CD4^+^ T cell targets [10], [12], [16], [17], [18]. This underscores the crucial role that metabolically activated CD4^+^ T-cells likely play in transmission[19].

Considering that the early stages of HIV infection represent a possible window of opportunity for preventing infection, the structural, functional and immunogenic characteristics of founder/early-transmitting gp120s are highly relevant to the design of a preventive HIV vaccine. These viruses invariably utilize CCR5, which is a key phenotype of early-transmitting gut-tropic isolates[20], [21]. The genotype of early-transmitting viruses has also been a subject of great interest. Although a genotypic signature of early-transmitting viruses has proved difficult to identify, two key features have emerged. In studies of both heterosexual and mother to child transmission, early-transmitting gp120s have been found to be shorter in length, and encode fewer potential N-linked glycosylation sites (PNGs) than typical chronically replicating isolates[11], [22], [23]. These features have thus far only been found in the context of infection with HIV subtypes A and C. Length shortening has been observed in the V1/V2 region, as well as V4 and flanking regions of gp120. PNGs absent from early-transmitting isolates (PNGΔs) appear in somewhat specific positions around the N- and C-terminal stems of V1/V2[11], [22] and in C3/V4[24]. These characteristics apparently provide early-transmitting isolates with increased transmission fitness[22], [24]; however, the nature of this fitness-advantage is unknown. The antigenic properties of early-transmitting isolates are the subject of ongoing studies [25], [26]. V1/V2, along with V4 and flanking regions are frequently an early target of autologous neutralizing antibodies (Nabs) [23], [27], [28]. The viral quasispecies escapes neutralization through amino acid substitutions, insertions/deletions (INDELs), and also by adding/shifting PNGs. In this way the bulk of the quasispecies drifts away from the genotypic features that distinguish early-transmitting isolates.

The V1/V2 domain of HIV-1 gp120 mediates binding to integrin α_4_β_7_ (α_4_β_7_) on CD4^+^ T-cells[29]. α_4_β_7_ has been termed the gut homing integrin[30]. It is upregulated on lymphocytes in Peyer's patches and mesenteric lymph nodes, and then mediates, in concert with chemokine receptors, the homing of these lymphocytes into GALT through interactions with its natural ligands, MadCAM and VCAM, which appear on gut endothelial cells[31]. α_4_β_7_ appears in close association with the CD4 receptor on mucosal CD4^+^ T cells[32]. The biochemical characteristics of the interactions between α_4_β_7_ and gp120 closely mimic those of α_4_β_7_ with MadCAM and VCAM. There is a strong dependence of this interaction on divalent cations, and the α_4_β_7_ binding-site in V1/V2 shares close sequence homology with the binding sites of α_4_β_7_'s natural ligands. This type of structural mimicry suggests that the specific affinity of gp120 for α_4_β_7_ provides increased fitness to HIV. However, unlike CD4 and CCR5, α_4_β_7_ is not required for viral entry or replication *in vitro*.

α_4_β_7_^+^/CCR5^+^/CD4^+^ memory T cells appear in the rectal and vaginal mucosa[32], [33]. In this way, this subset of CD4^+^ T cells links the portal of entry during sexual transmission and the inductive and effector sites of the gut that provide a permissive environment for near-exponential replication. We have proposed that HIV evolved a specific affinity for α_4_β_7_ as a means of insuring that productive target cells with gut-homing potential will be infected shortly after transmission. We noted, however, that the α_4_β_7_-reactivity of gp120s varied widely among isolates we analyzed [29], suggesting to us that strong α_4_β_7_-reactivity might provide increased transmission fitness over those isolates with lower α_4_β_7_ –reactivity in the context of mucosal transmission.

In the present study we provide evidence that the apparent selection at the time of mucosal transmission for viral envelopes exhibiting transmission-linked PNGΔs coincides with dramatic increases in α_4_β_7_-reactivity. We conclude that α_4_β_7_-reactivity is one of the phenotypes that can contribute to the genetic restriction that occurs during mucosal transmission.

## Results

### Transmission linked PNGΔs in gp120 increase affinity for α_4_β_7_

**Figure 1:**
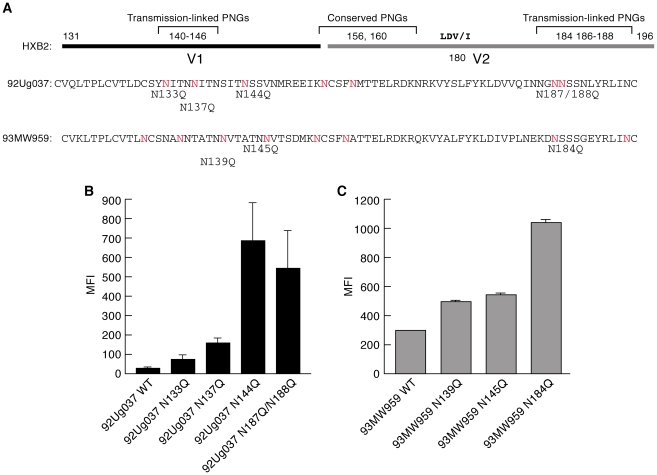
Effect of V1/V2 PNGΔs on the α_4_β_7_-reactivities of a subtype A and a subtype C gp120. A) The V1/V2 regions of 92Ug037 (subtype A) and 93MW959 (subtype C) aligned with HXBII V1/V2. PNGΔs associated with transmission are designated using HXBII numbering. PNGs that appear in 92Ug037 and 93MW959 are highlighted in red. PNGΔs introduced into each protein by N/Q substitution appear directly below. B) α_4_β_7_-reactivities of 92Ug037 w.t. gp120 and PNGΔ mutants are reported as mean fluorescence intensity (MFI). C) α_4_β_7_-reactivities of 93MW959 w.t. gp120 and PNGΔ mutants. Error bars report the standard deviation between two replicates, and results are representative of three independent experiments using independent donor CD4^+^ T cells.

Longitudinal studies of cohorts of couples discordant for HIV infection have been used to isolate and characterize genotypic, phenotypic and immunogenic properties of early-transmitting gp120s. In studies involving heterosexual transmission of subtype A and C viruses, early-transmitting viruses tend to encode more compact variable loops of gp120 with reduced numbers of PNGs relative to isolates replicating during the chronic phase of infection[11], [22], [23]. This pattern represents a bias rather than a rule, and although it is most frequently associated with the V1/V2 domain of gp120, the V4 and flanking regions of early replicating viruses can also display these characteristics. In the V1/V2 domain, transmission-linked PNGΔs are clustered at the N- and C-termini of V1 and V2 respectively, while two central PNGs are well conserved (Figure 1A). The apparent advantage that these characteristics confer upon early-transmitting viruses is not known. The V2 PNGs lie close to a tripeptide (LDV/I – Figure 1A) that mediates gp120 binding to α_4_β_7_ [29]. We determined whether removing PNGs in V1/V2 altered reactivity with α_4_β_7_. Two recombinant gp120s, 92Ug037 (subtype A), and 93MW959, (subtype C), were employed. Both envelopes were derived from viruses obtained from asymptomatic females who contracted HIV-1 through heterosexual transmission. 93MW959 was isolated ∼12 months post-seroconversion, while the time between seroconversion and isolation of 92Ug037 is unknown. PNGs were removed by site-directed mutagenesis in which asparagines were replaced with glutamines (Figure 1A). α_4_β_7_-reactivity was measured using a steady-state binding assay that employs primary α_4_β_7_^+^/CD4^+^ T-cells (Figure S1). Each PNG mutant was compared to its wild type (w.t.) parent. For 92Ug037, PNGΔs near the N-terminus of V1 or the C-terminus of V2 mediated increases in α_4_β_7_-reactivity of up to 20-fold (Figure 1B). For 93MW959, a single PNGΔ at the C-terminus of V2 mediated a ∼3.5-fold increase in α_4_β_7_-reactivity, while PNGΔs near the N-terminus of V1 had little effect on α_4_β_7_-reactivity (Figure 1C).

**Figure 2:**
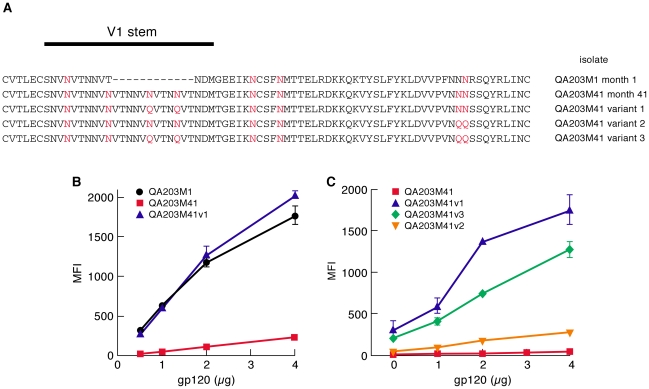
A comparison of the α_4_β_7_-reactivities mediated by the V1/V2 regions of a month-1 gp120 and month 41 gp120 isolated from a single patient. A) The gp120 V1/V2 sequences from patient QA203 obtained at month 1 and month 41 are listed. PNGs are highlighted in red. Variants, 1, 2 and 3 of QA203M41 are included with the N/Q substitutions highlighted in red. B) α_4_β_7_-reactivities of chimeric gp120s encoding the V1/V2 regions of QA203M1, QA203M41 and QA203M41 variant 1 are reported as mean fluorescence intensity. C) A comparison of the α_4_β_7_-reactivities of variants 1, 2 and 3 of QA203M41. QA203M41 is included for reference. Error bars represent the standard deviation of two replicates, and results are representative of three independent experiments using independent donor CD4^+^ T cells.

Length-shortening in V1/V2 is also associated with early transmitting gp120s. However, because those deletions often result in PNGΔs, it is difficult to determine whether short V1/V2s are favored independently of PNGΔs [34]. We examined the influence of V1/V2 length shortening on α_4_β_7_-reactivity by taking advantage of a previously characterized pair of subtype A envelopes isolated from an individual at ∼1-month and ∼41-months post-infection[23]. The month 1 V1/V2 (QA203M1) encodes 63 amino acids and 5 PNGs, while the month 41 V1/V2 (QA203M41) encodes 70 amino acids and 8 PNGs such that the 41 month envelope encodes two additional PNGs near the N-terminus of V1 and one additional PNG near the C-terminus of V2 (Figure 2A). Both V1/V2s were grafted into a subtype A gp120 backbone isolated ∼1year post-infection from a second patient. QA203M1 gp120 displayed ∼20× greater α_4_β_7_-reactivity than did w.t. QA203M41 (Figure 2B). To distinguish the influence of V1/V2 length from the influence of the number of PNGs on α_4_β_7_ reactivity we constructed a variant of the month-41 V1/V2 (QA203M41variant1) lacking the two V1 PNGs that were missing in QA203M1 without changing its length (Figure 2A). QA203M41 variant 1, exhibited relatively strong binding to α_4_β_7_ that was nearly identical to that of QA203M1 gp120. These results indicate that increased α_4_β_7_-reactivity mediated by the early-transmitting QA203M1 relative to QA203M41 was due to PNGΔs rather than to a shorter V1/V2. We extended this analysis by removing additional PNGs from QA203M41. QA203M41 variant 2 in which PNGs were removed from the C-terminal region of V2 mediated a small increase in α_4_β_7_-reactivity, while QA203M41 variant 3, which combines variant 1 and variant 2 PNGΔs mediated an increase in α_4_β_7_-reactivity that was intermediate between variant 1 and variant 2 (Figure 2A, C). These results demonstrate that PNGΔs do not necessarily enhance α_4_β_7_-reactivity in an additive manner.

Taken together, analysis of all of the PNGΔs described above leads us to conclude that the removal of transmission-linked V1/V2 PNGs can mediate large increases in the α_4_β_7_-reactivity of both subtypes A and C gp120s. The pattern in which these increases were mediated is complex such that no single PNGΔ at a given position in V1/V2 mediated increased α_4_β_7_-reactivity in all three gp120s. PNGΔs near both the N- and C-termini of the V1/V2 of 92Ug037 increased α_4_β_7_-reactivity, while only one PNGΔ near the C-terminus of the V2 of 93MW959 increased α_4_β_7_-reactivity. For QA203M41, the opposite was the case, removal of V1 PNGs, but not V2 PNGs mediated a large increase in α_4_β_7_-reactivity. These data suggest that increased α_4_β_7_-reactivity mediated by PNGΔs is due to changes in V1/V2 conformation rather than through steric occlusion, a subject that will be addressed below.

**Figure 3:**
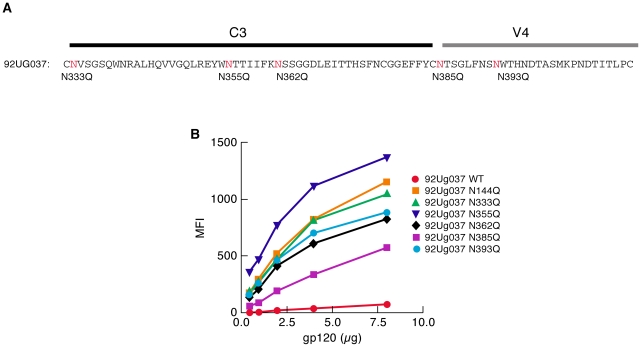
Effect of PNGΔs in the C3/V4 region on the α_4_β_7_-reactivity of 92Ug037 gp120. A) The positions of transmission-linked PNGs in the C3/V4 region of subtype A gp120s are highlighted in red in the C3/V4 region of 92Ug037 and N/Q substitutions are listed directly below. B) α_4_β_7_-reactivity of each 92Ug037 PNGΔ mutant was compared to w.t 92Ug037 gp120 and reported as mean fluorescence intensity (MFI) 92Ug037N144Q was included for reference. Results are representative of three independent experiments using independent donor CD4^+^ T cells.

A study of early-transmitting viruses following mother to child transmission reported that PNGs in the C3/V4 region of gp120 are also underrepresented in early replicating isolates in a manner similar to that described above for V1/V2 PNGs [24]. Overbaugh and colleagues found that PNGs at specific positions throughout C3/V4 were underrepresented in early-transmitting isolates of subtype A viruses isolated from infants shortly after birth. Although this region of gp120 is far removed from the known α_4_β_7_-binding site in V1/V2, we determined whether removing these PNGs would also increase α_4_β_7_-reactivity. The C3/V4 region of 92Ug037 was aligned with subtype A sequences from the study noted above and five transmission-linked 92Ug037 PNGΔ gp120s were analyzed (Figure 3A). 92Ug037N333Q, 92Ug037N362Q, and 92Ug037N393Q gp120s mediated increases in α_4_β_7_-reactivity of ∼18 to 21-fold. Interestingly, Ug037N355Q mediated a ∼27-fold increase in α_4_β_7_-reactivity. This increase was greater than that mediated by Ug037N144Q, which among the 92Ug037 V1/V2 PNGΔs mediated the largest increase in α_4_β_7_-reactivity. 92Ug037N385Q mediated an ∼8-fold increase in α_4_β_7_-reactivity (Figure 3B). We conclude that, as with V1/V2, PNGΔs in C3/V4 can mediate large increases in α_4_β_7_-reactivity. The increased α_4_β_7_-reactivity achieved by PNGΔs in C3/V4 supports the proposition that the manner in which glycans inhibit gp120 binding to α_4_β_7_ involves conformational changes in gp120 rather than simple steric occlusion. Data supporting this interpretation will be presented below.

### Glycan type impacts gp120- α_4_β_7_ interactions

**Figure 4:**
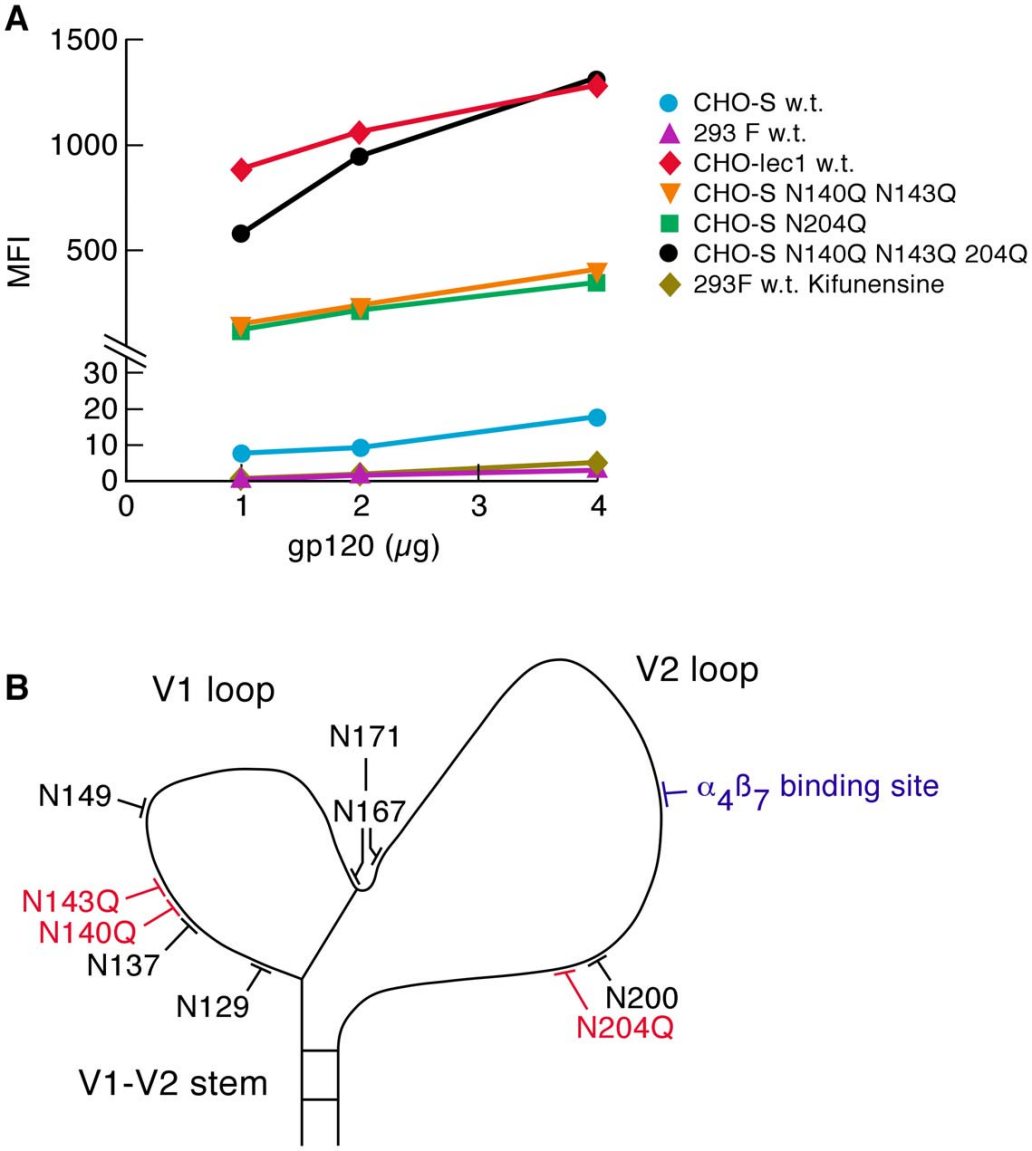
Effect of glycan content on the α_4_β_7_-reactivity. A) α_4_β_7_-reactivity of the ancestral AN1 gp120 (subtype B) produced in CHO-S cells, 293F cells, CHO lec1 cells and kifunensine treated 293F cells, were compared and reported as mean fluorescence intensity (MFI). AN1 V1/V2 PNGΔs were also included. Results are representative of three independent experiments using independent donor CD4^+^ T cells. B) Positions of all PNGs and the α_4_β_7_ binding site in the V1/V2 loop of AN1 gp120 are illustrated.

To better understand the role of glycan deletion in gp120-α_4_β_7_ interactions we compared the α_4_β_7_-reactivity of AN1 w.t. gp120, a subtype B ancestral/consensus gp120[35], expressed in three different cell lines known to glycosylate gp120 in different ways. AN1 w.t. was expressed in CHO S cells (a nonadherent subclone of CHO K1), the same cell line in which all of the gp120s described in the present study thus far were produced. gp120s expressed in CHO K1 cells present a heterogeneous pattern of oligo-mannose and complex carbohydrate type glycans [36]. Complex carbohydrates tend to appear on the solvent-exposed loops, including V1/V2 of recombinant gp120 proteins [37]. We also expressed AN1 gp120 in CHO lec1 cells, a CHO derivative that lacks *N*-acetylglucosamine (GlcNAc) glycosyl transferase activity, so that N-linked carbohydrate trimming is blocked at the Man5-GlcNAc2-Asn intermediate (where Man is Mannose)[38]. gp120s produced in CHO lec1 cells are devoid of complex carbohydrate, and are instead enriched with oligo-mannose type glycans. Finally, we expressed AN1 w.t. gp120 in 293F cells (a nonadherent subclone of HEK 293T cells), which differ from both CHO cell lines in the manner in which it modifies complex carbohydrate. 293T derived cells sialylate the terminal galactose moieties of complex carbohydrates through both α-2,3 and α-2,6 linkages. CHO cells, which lack α-2, 6-sialyltransferase, establish these linkages only at the α-2,3 position [39]. CHO S expressed AN1 w.t. gp120 reacted with α_4_β_7_ at an intermediate-low level (Figure 4A), similar in magnitude to many of the gp120s that we have previously reported[29]. CHO lec1-derived AN1 w.t. gp120 reacted ∼100× more efficiently with α_4_β_7_ than did CHO S AN1 w.t. gp120 (Figure 4A). It is likely that this increase in α_4_β_7_-reactivity results from the substitution of complex carbohydrates in V1/V2 with oligo-mannose type glycans. In contrast, 293F expressed AN1 w.t. gp120 showed no detectable α_4_β_7_-reactivity. We subsequently analyzed six additional 293F and T derived gp120s, including CAP881m.c17, a gp120 that exhibits very strong α_4_β_7_-reactivity when derived in CHO S cells, and without exception they all exhibited low or undetectable α_4_β_7_-reactivity (Figure S2 and data not shown). Considering the differential sialylation mediated by CHO S and 293 cells, we digested 293F AN1 w.t. gp120 with neuraminidase, which catalyzes the hydrolysis of terminal sialic acid residues; however, this failed to rescue α_4_β_7_-reactivity (data not shown). We next expressed AN1 w.t. gp120 in 293F cells cultured in the presence of kifunensine, a mannosidase I inhibitor that restricts the processing of N-linked glycosylation beyond the Man_9_GlcNac_2_ intermediate and swainsonine, a mannosidase II inhibitor that restricts the processing of N-linked glycosylation beyond hybrid glycan intermediates [40]. Expressing gp120 in 293F cells in the presence of these drugs should, to some degree approximate the complex carbohydrate deficient glycan characteristics of CHOlec1 derived gp120. Neither drug rescued α_4_β_7_-reactivity (Figure 4A and data not shown). Although we do not know what the post-translational defect is in 293F and 293T-expressed gp120s, we conclude that utilizing gp120s produced in this manner may not be ideal for studies involving α_4_β_7_.

We next determined what effect restricting the glycans on AN1 w.t. gp120 to the oligo-mannose type, as occurs in CHO lec1 cells, would have compared to the effect of the deletion of transmission-linked glycans in AN1 derived from CHO K1 cells. Three AN1 PNGΔ derivatives: AN1 NN140/143QQ, AN1 N204Q, and the combined mutant AN1 NN140/143QQ,N204Q, were expressed in CHO-S cells (Figure 4B). These PNGs appear near the N- and C-termini of V1 and V2 and their positions correspond to transmission-linked PNGs. Compared to AN1 w.t. expressed in CHO-S cells, AN1 NN140/143QQ increased α_4_β_7_-reactivity by ∼25-fold (Figure 4A). AN1 N204Q increased α_4_β_7_-reactivity by ∼23-fold. The combined mutant NN140/143QQ,N204Q increased α_4_β_7_-reactivity by ∼96-fold, and resulted in a CHO-S derived gp120 with α_4_β_7_-reactivity ∼equivalent to CHO lec1 gp120. To formally demonstrate that increased α_4_β_7_-reactivity can result from reduced N-linked glycosylation we carried out a brief digestions of CHOlec1 derived AN1 gp120 with endoglycosidase H (Endo-β-N-acetylglucosaminidase H) and determined that the enzymatic removal of PNGs from AN1 gp120 leads to increased α_4_β_7_-reactivity (Figure S3). Overall these results demonstrate that different glycosylation patterns mediated by different cells can exert a strong influence on α_4_β_7_-reactivity. In the case of AN1 gp120, replacing complex carbohydrate with oligo-mannose type glycans mediates stronger α_4_β_7_-reactivity, but this same strong binding can be achieved by deleting a small number of PNGs in V1/V2.

### α_4_β_7_ –Reactivity and escape from autologous neutralizing antibodies

A pseudotyped virus encoding the month 1 QA203M1 V1/V2, described above (Figure 2), was efficiently neutralized by autologous serum taken at month 40, while a pseudotyped month 41 QA203M41 virus escapes neutralization from this same serum[23], indicating that neutralization-escape in the 40 month isolate was mediated by V1/V2. This pattern of escape is somewhat typical of early- vs. chronic-replicating HIV-1 viruses and reflects the fact that in subtype A and C viruses V1/V2 is frequently a direct target of early autologous neutralizing antibodies[23], [27], [28]. Additionally, V1/V2 can mediate a second type of neutralization-escape that affects epitopes throughout gp120. Mutations in V1/V2 can enhance a structural property of gp120 that has been termed conformational masking[41], [42], [43], [44]. Both mechanisms of escape are mediated by a combination of sequence variation, INDELS, and the addition and/or position-shifting of PNGs. We questioned whether changes in gp120 leading to neutralization-escape would disrupt α_4_β_7_-reactivity. Two recent studies [27], [28], in which quasispecies evolution and autologous neutralizing Ab responses were followed longitudinally, beginning shortly after transmission, provide perhaps the best-defined examples of V1/V2-mediated escape. Rong, Derdeyn and colleagues[28], demonstrated that the antibodies present in an HIV-1 subtype C infected female patient (patient 205F) at ∼38 months post-infection neutralized the early-replicating founder virus isolated within the first month following sexual transmission. Importantly, virtually all of the neutralizing activity in the 38-month sera targeted V1/V2 dependent epitopes. In a second study Moore, Morris and colleagues describe a more complex pattern of neutralization-escape[27], in which the early-transmitting month 1 virus isolated from a female patient, CAP88 (subtype C), was sensitive to autologous serum taken after 13 months, while viruses replicating at month 12 were not. Month 12 isolates were able to escape the month 13 sera via INDELS and PNG additions in both V1/V2 and C3.

**Figure 5:**
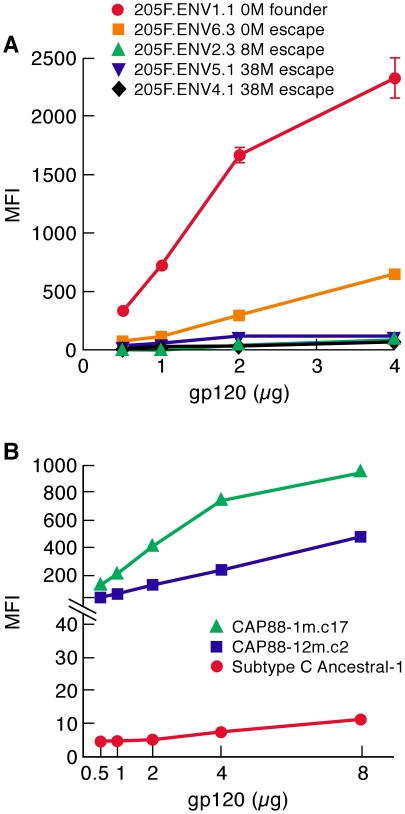
α_4_β_7_-reactivity of early-transmitting gp120s. A) gp120s corresponding to patient 205F 0 month founder, 0 month escape, 8 month escape and two 38 month escape viruses were assayed for α_4_β_7_-reactivity and reported as mean fluorescence intensity (MFI). B) gp120s corresponding to a 1 month virus and a 12 month virus isolated from patient CAP88 were assayed for α_4_β_7_-reactivity and reported as mean fluorescence intensity (MFI). A consensus/ancestor subtype C gp120 was included for reference. Error bars represent the standard deviation of two replicates, and results are representative of three independent experiments using independent donor CD4^+^ T cells.

We produced the neutralization sensitive 205F 0-month founder gp120 (Z205F.ENV1.1), and four escape mutants. The 0- and 8- month escape viruses are highly sensitive to neutralization by 38-month plasma and this escape was mediated by sequence changes that lie entirely within V1/V2 (Figure S4). The two 38-month viruses are partially resistant to neutralization by 38-month autologous plasma. The 205FENV1.1 0-month founder exhibited ∼8-fold greater α_4_β_7_–reactivity than did the 0-month escape, and at least 17-fold greater than did the 8- and 38-month escape gp120s (Figure 5A). The level of α_4_β_7_-reactivity exhibited by the 205F.ENV1.1 0-month founder appeared to be substantially greater than many of the w.t. gp120s we previously reported[29]. We conclude that the high level of α_4_β_7_ reactivity in the 0-month founder was lost concomitant with sequence changes that mediated neutralization-escape.

We next compared the α_4_β_7_-reactivity of CAP88 gp120s isolated at 1 month and 12 months post infection. We chose CAP88.1m.c17, which reflects the predominant circulating isolate early in the first month post-infection. We also expressed the 12 month isolate CAP88.12m.c2 that diverged from CAP88.1m.c17 by adding PNGs in both V1/V2 and C3 at positions that correspond to transmission-linked glycans (Figure S4). The addition of these PNGs was shown to contribute to the resistance of CAP88.12m.c2 to autologous serum taken after month 13[27]. Like the 205F 0 month founder, CAP88.1m.c17 exhibited strong α_4_β_7_ reactivity (Figure 5B). Surprisingly this high level of reactivity was maintained in CAP88.12m.c2 despite the fact that PNGs were added at transmission-linked PNG positions in both V1/V2 and C3. This result demonstrates that viruses with relatively strong α_4_β_7_-reactivity can escape from autologous neutralizing antibodies without losing their α_4_β_7_-reactivity. Considering the complex pattern of changes in α_4_β_7_-reactivity we observed with the various N/Q substitutions described in Figures 1, 2 and 4, in which no single PNGΔ generated the same effect on each of the proteins analyzed, we conclude that the glycans that can mediate neutralization-escape may impact α_4_β_7_ reactivity (e.g. 205F), but not in all gp120s, and not in a way that is easily predictable.

In summary, we find that both of the early-replicating gp120s we analyzed showed high levels of α_4_β_7_-reactivity. Escape from neutralizing antibodies disrupted this activity in gp120s derived from patient 205F, but the α_4_β_7_-reactivity of a gp120 derived from patient CAP88 persisted 12 months post-infection despite sequence changes in V1/V2 and C3 that mediated escape from autologous neutralizing antibodies.

### α_4_β_7_-Reactivity in relation to CD4 affinity

**Figure 6:**
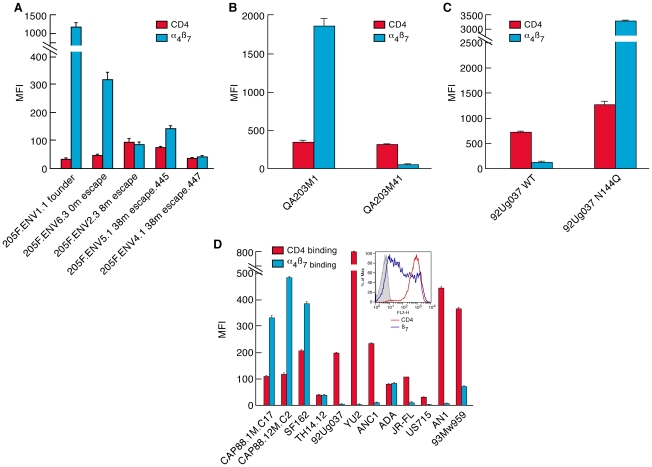
A comparison of steady-state CD4-reactivity and α_4_β_7_-reactivity of a panel of gp120s. A) Levels of CD4-reactivity (red) and α_4_β_7_-reactivity (blue) were assessed for each patient 205F gp120, B) the QA203 month 1 and month 41 chimeric gp120s, C) w.t. and N144Q 92Ug037 gp120s, D) the 1-month and 12 month CAP88 gp120s and a panel of well characterized gp120s including an ancestral B (AN1) and an ancestral C (ANC1) gp120. Inset indicates the staining of the cells employed in this assay with PE conjugated CD4 (red) and β7 (blue) mAbs. Error bars represent the standard deviation of two replicates, and results are representative of three independent experiments using independent donor CD4^+^ T cells.

The affinity of HIV-1 gp120s for CD4 varies over a wide range and these differences can influence the cell-tropism of a viral isolate. For example, high CD4 affinity facilitates replication in macrophages by compensating for the low density of CD4 appearing on the macrophage membrane[45]. Changes in CD4 affinity could theoretically impact the transmissibility of a viral isolate; however, studies of early replicating gp120s have not, to date, found any clear correlation between CD4 affinity and transmission fitness [25]. Numerous studies have, however, shown that both amino acid substitutions and glycan additions/deletions in V1/V2 can influence the conformation of gp120 in a global way [44], [46]. Because V1/V2 plays an important role in α_4_β_7_ recognition we determined whether there was any relationship between α_4_β_7_-reactivity and CD4-reactivity. We first employed a steady-state binding assay to compare the reactivity of gp120s for α_4_β_7_ and CD4. In this assay, retinoic acid-cultured CD4^+^ T cells were differentially masked with either a CD4 mAb or an α_4_ mAb, as described in supporting Figure S1. For gp120s derived from early-transmitting viruses we found that α_4_β_7_ mediated a greater degree of binding to the cell surface than did CD4. The amount of 205F 0-month founder gp120 bound to α_4_β_7_ was 37-fold greater than that bound to CD4, but this differential disappeared in each of the 205F escape gp120s (Figure 6A). Such differences could be mediated entirely by V1/V2, as demonstrated by comparing the CD4- and α_4_β_7_-reactivities of the chimeric QA203M1 and QA203M41 gp120s, which are identical in all domains other than V1/V2. 1-month QA203M1 bound 5-fold more to α_4_β_7_ than to CD4 while the 41 month QA203M41 was captured primarily by CD4 (Figure 6B). The deletion of a single transmission-linked PNG was sufficient to achieve a binding profile in which more binding to the cell membrane was mediated by α_4_β_7_ than by CD4. For example, while the majority of w.t. 92Ug037 binding to the cell surface was mediated by CD4, PNGΔs at the N terminus of V1/V2 altered this pattern such that more binding was now mediated by α_4_β_7_ (Figure 6C). Finally, we compared the two CAP88 gp120s to a panel that included several widely studied gp120s and subtypes B and C ancestral/consensus gp120s[35], [47]. Similar to the 205F 0 month founder and the chimeric QA203M1, both CAP88 proteins showed preferential binding to α_4_β_7_ over CD4 (Figure 6D). In contrast to the CAP88 gp120s, all other gp120s in the panel, with one exception, exhibited CD4 binding that was equal to or greater than α_4_β_7_ binding. The one exception was SF162, a highly neutralization-sensitive gp120 whose gut tropic characteristics have been well-documented[20], [21]. Because levels of α_4_β_7_ expression on the cells we employed were equivalent to, or less than CD4 expression levels (Figure 6 inset and Materials and Methods) we can conclude that the steady-state affinity of gp120s, like the two CAP88 proteins, for Mn^++^ activated α_4_β_7_ is greater than their steady-state affinity for CD4. In addition, this comparison underscores the strong α_4_β_7_-reactivity of the early-replicating gp120s we analyzed, relative to a number of well-studied gp120s. In this way, these gp120s appear to be better adapted to interact with CD4^+^ T cells that express a gut-homing receptor.

**Figure 7:**
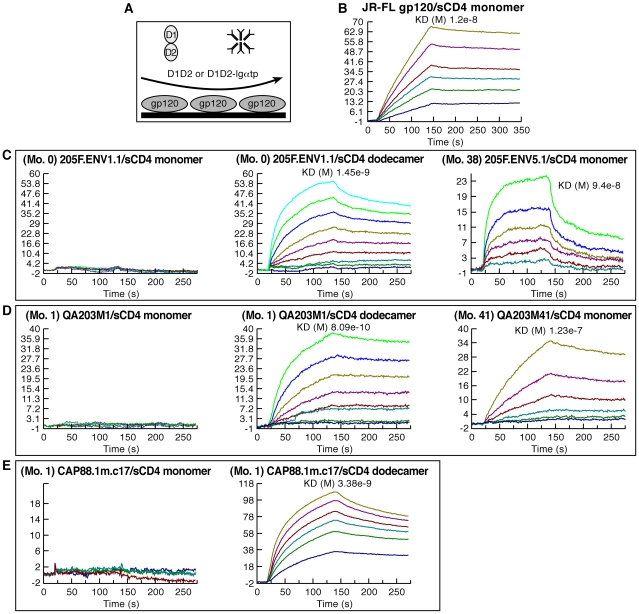
CD4 binding characteristics of early-transmitting gp120s. A) Schematic of the Surface Plasmon resonance analysis strategy used to measure the reactivity of monomeric sCD4 (D1D2) and dodecameric sCD4 (D1D2-Igαtp). B–E) Sensorgrams depicting the binding kinetics of increasing concentrations of D1D2 or D1D2-Igαtp reacting with immobilized gp120s, the identities of which are noted in each panel. Each ligand/analyte pair is listed and overall affinity is reported as K_D_ (where applicable). These results are representative of three independent experiments using independently coupled gp120 biosensor surfaces.

It is noteworthy that the large changes in steady-state binding to α_4_β_7_, mediated by sequence changes in V1/V2, were coupled with relatively small changes in steady-state binding to CD4 (Figure 6). In some HIV-1 isolates, insensitivity to sCD4 and CD4 binding-site antibody neutralization is mediated by V1/V2 [44], [46], a phenomenon termed conformational masking[41], [42], [43]. Of note, this masking-effect can be modulated by the same transmission-linked PNGs that exert a strong influence on α_4_β_7_-reactivity [44], [46]. To better understand the relationship between CD4-reactivity, V1/V2 masking, and α_4_β_7_-reactivity we employed a surface plasmon resonance-based kinetic CD4 binding assay. gp120s were immobilized on the surface of a biosensor chip and either monomeric sCD4 (D1D2), or D1D2-Ig αtp, a highly oligomerized (dodecameric) CD4-Ig derivative[48], [49], [50], was passed over the surface, allowing us to measure reaction kinetics (Figure 7A). In this format D1D2-Ig αtp can bind >1 gp120 in a near-simultaneous manner, allowing avidity-effects to contribute to interactions between it and gp120. However, in this format, avidity cannot contribute to monomeric sCD4-gp120 interactions. Sensorgrams of nine well-studied gp120s, including JR-FL gp120 (Figure 7B), and eight additional gp120s (Figure S5) are provided for reference. 205FENV1.1 0-month founder gp120 failed to recognize monomeric CD4, but did exhibit high affinity for dodecameric D1D2-Igαtp (Figure 7C). The failure of this gp120 to react with monomeric CD4 distinguishes it from the nine standard gp120s we analyzed (Figure 7B and Figure S5). However, we observed the same phenomenon with the1-month QA203M1 chimeric gp120 (Figure 7D), and CAP88 1m.c17(Figure 7E). In patient 205F and QA203 reactivity with monomeric sCD4 reappeared as the viral quasispecies evolved away from the early transmitting isolate. Both the 38 month 205F.ENV5.1 gp120 (Figure 7C) and 41 month QA203M41(Figure 7D), which bind weakly to α_4_β_7_, did bind monomeric sCD4 with high-affinity. In summary, all three early-transmitting gp120s failed to react with monomeric sCD4, which distinguishes them from nine standard gp120s. Because the chimeric QA203M41 and QA203M1 gp120s differ only in V1/V2 we conclude that the failure of QA203M1 to bind monomeric sCD4 was mediated by V1/V2, and it seems likely that this also holds for 205F.ENV1.1 0-month founder, and CAP88 1m.c17. All three of these gp120s did, however, react with dodecameric D1D2-Igαtp (Figure 7 C, D, E), and with CD4 displayed on the surface of a T cell (Figure 6). The failure to interact with monomeric sCD4 in this SPR based assay does not indicate that the viruses from which these gp120s were derived are CD4-independent, nor can one conclude that these viruses would be resistant to sCD4 neutralization. This observation does indicate that the CD4-reactivity of these early-replicating gp120s is more dependent on avidity-effects than is the case for the other gp120s that we analyzed, and further suggests that, in addition to high-level α_4_β_7_-reactivity, these gp120s share structural features that distinguish them from many gp120s. Although these distinguishing biochemical features involve gp120 monomers, it is reasonable to suggest that these features will in some manner impact the stability and immunogenicity of trimeric spikes[51].

## Discussion

The search for a consistent genotypic signature of early-transmitting viruses has proven difficult. The most consistently observed genotypic marker thus far identified is a more compact V1–V4 with a reduction in the number of PNGs, relative to the length and average number of PNGs in chronically replicating isolates[11], [22], [23]. Early-transmitting viruses bearing this genotype do not appear to use CD4 or CCR5 in any way that distinguishes them clearly from chronically replicating isolates[25], [52]. However, we found that removing transmission-linked PNGs from multiple gp120s consistently resulted in greatly increased α_4_β_7_-reactivity. Additionally, the early-transmitting gp120s that we analyzed showed notably higher levels of α_4_β_7_-reactivity than many of the chronically replicating isolates that we assayed. These results suggest that increased α_4_β_7_-reactivity is likely to be part of the phenotype underlying PNGΔs in some early- transmitting viruses and that this increased reactivity provides, under certain conditions, increased fitness in the process of transmission. Under this scenario, engaging α_4_β_7_ could provide an advantage at an early stage of transmission, but may not always be required. We should emphasize that additional analyses, that include greater numbers of early-transmitting gp120s are needed to better estimate the importance of α_4_β_7_^+^/CD4^+^ T-cells in mucosal transmission. Moreover, we do not yet know the specific point in the process of transmission where α_4_β_7_-reactivity may have an impact, nor do we know the relative importance of α_4_β_7_-reactivity on transmission fitness relative to other phenotypic features of gp120. However, the fact that HIV has evolved an affinity for α_4_β_7_ reflects the important role that α_4_β_7_^+^/CD4^+^ T-cells likely play in the process of mucosal transmission. This is of potential importance since it follows logically that blocking viruses from infecting these cells will reduce the frequency of successful transmission.

We analyzed only two early-transmitting gp120s and one early-transmitting chimera. Clearly, we cannot generalize from this small number, yet it is noteworthy that all three envelopes bind more efficiently to activated α_4_β_7_ than to CD4. Thus, these gp120s are better adapted to interact with α_4_β_7_^+^/CD4^+^ T cells as opposed to α_4_β_7_^lo-neg^/CD4^+^ T cells. We previously reported that metabolically activated cells are enriched in the α_4_β_7_^+^ subset of CD4^+^ T cells in rectal mucosa[32], and a similar observation has been made for cervical α_4_β_7_^+^/CD4^+^ T cells (R. Kaul, personal communication). CD4 is not a marker of cellular activation or of gut-homing. When the affinity of a gp120 for CD4 is high, e.g. JR-FL gp120, it engages all CD4 cells regardless of their metabolic state or homing potential. CCR5 expression can correlate with metabolic activity; however, prior to CD4-binding it is hidden from HIV. By increasing α_4_β_7_-reactivity, which is CD4-independent, we find that the early-transmitting gp120s that we analyzed are better adapted to engage with a specific subset of CD4^+^ T cells that are highly susceptible to infection by both HIV and SIV[32], [53], and located in anatomical sites relevant to HIV transmission. The context in which the interaction between HIV-1 and α_4_β_7_^+^/CD4^+^ T cells takes place remains unclear. It may involve cell-free virions, but it might also occur in the context of dendritic cell-CD4^+^ T cell interactions that are potentially important in mucosal transmission[54]. With one exception (SF162 gp120), none of the commonly studied gp120s that we characterized here or in our previous report exhibited this phenotype[29]. Although these standard gp120s may not necessarily be representative of chronic HIV isolates, it is noteworthy that several showed near-undetectable α_4_β_7_-reactivity. This is consistent with our previous observation that α_4_β_7_ interactions are not required for viral replication[29] and underscores the fact that α_4_β_7_-reactivity can diminish rapidly in a newly infected individual (e.g. patient 205F). We note, however, that the introduction of a small number of transmission-linked PNGΔs into a gp120 derived from a chronically replicating virus (e.g QA203M41 variant 1) can generate a strong α_4_β_7_-reactive phenotype such that this gp120 is now better adapted to engage CD4^+^ T cells that express α_4_β_7_. Because these transmission-linked PNGΔs have been found in a somewhat consistent manner in early-transmitting gp120s in multiple studies involving transmission of subtypes A and C viruses [11], [22], [23], [24], [55], it will be important in future studies to determine the extent to which strong α_4_β_7_-reactivity is a phenotype that is overrepresented in early-transmitting isolates. It will also be important to determine the frequency with which early-replicating viruses bind more efficiently to activated α_4_β_7_ than to CD4.

The frequency and circumstances under which viruses encoding fewer PNGs in V1–V4 are preferentially established in a newly infected individual provides clues concerning the possible role of gp120-α_4_β_7_ interactions in facilitating mucosal transmission. We note that transmission-linked PNGΔs have thus far been found only in cohorts of heterosexual couples discordant for HIV infection and a mother to child transmission cohort, all of which involve subtypes A and C, but not B[56]. The failure to observe this genotypic pattern in subtype B cohorts may reflect differences in the predominant modes of transmission i.e. heterosexual transmission versus transmission among men who have sex with men and intravenous drug users. It may also reflect other acquisition related risk factors, such that gp120-α_4_β_7_ interactions may play a greater role under conditions in which the risk of acquisition is low, but less of a role when that risk is high. One risk factor that can influence susceptibility to infection is the presence of STDs that cause inflammation of genital tissues in a recipient [10], [12], [16], [17], [18]. To the extent that inflammation increases the availability of activated CD4^+^ T cells near the site of infection, the selection pressure for a virus with strong α_4_β_7_-reactivity may be diminished. However, an opposite dynamic may also be operative under different circumstances. *Chlamydia* infection (and possibly other STDs) in the female genital tract has been shown to increase the number of antigen-specific α_4_β_7_^+^ T cells migrating through the female genital tract and into GALT[33], [57], [58], [59]. Under these conditions, isolates that can bind α_4_β_7_ are more likely to engage activated CD4^+^ T cells. Further phenotypic characterization of α_4_β_7_^+^ T cells in mucosal tissues, as well as the early-transmitting viruses derived from different types of transmission cohorts, will be necessary to clarify the relationship between acquisition risk factors and α_4_β_7_-reactivity.

We found that the type of glycans that decorate gp120 can exert a strong influence on α_4_β_7_-reactivity. By producing gp120 in a cell that fails to process oligomannose glycans into larger complex glycans we were able to increase the α_4_β_7_-reactivity of AN1 gp120 by >100-fold. In this regard, it is well established that different cell types, and even the same cell type, under different metabolic conditions, glycosylate proteins differently [60], [61]. We know that gp120s produced in macrophages are glycosylated differently than the same gp120s produced in CD4^+^ T cells[62]. It follows then that α_4_β_7_-reactivity, and possibly the transmissibility of a virion, may be influenced by the type of cell in which that virion is produced *in vivo*. It would be useful if we could tailor our *in vitro* gp120 expression systems in a way that reflects *in vivo* glycosylation; however, although a recent study has characterized the glycan content presented on virion-associated spikes generated in primary PBMCs *in vitro* [63], we do not yet know the true glycan profile of any *in vivo* derived HIV-1 gp120. In addition to glycosylation, other post-translational modifications may also influence α_4_β_7_-reactivity. We found that gp120s produced in 293F and 293T cells showed greatly reduced α_4_β_7_-reactivity, which could not be explained entirely by differences in glycosylation. These observations may prove useful in evaluating the limitations of 293-derived gp120 for transmission-related studies. Overall these data indicate that post-translational modifications in gp120 have the potential to influence the transmissibility of HIV-1, and that the type of carbohydrate that appears on an envelope may play a critical role in the selection of early-transmitting isolates.

Although certain PNGs in V1/V2 may sterically interfere with gp120 binding to α_4_β_7_, the manner in which transmission-linked PNGΔs throughout V1–V4 increased α_4_β_7_-reactivity suggests that increased α_4_β_7_-reactivity resulted from a change in the conformational state(s) of gp120. In this regard, it is well established that V1-V4 glycans can influence gp120 conformation globally[64], [65]. In addition, it is noteworthy that the naturally occurring gp120s that we analyzed exhibited either strong or weak α_4_β_7_-reactivity, since it suggests that there is a distinct conformational or structural feature associated with some early-transmitting gp120s that allows them to engage α_4_β_7_ in an efficient way. It is highly likely that sequence changes independent from PNGs will also influence α_4_β_7_-reactivity. In fact, the relatively high level of α_4_β_7_-reactivity exhibited by the 205F 0-month founder gp120 when compared to later isolates from this patient cannot be explained by a reduced number of PNGs. This underscores the limitation of using a genotypic feature i.e. the number of PNGs, to predict a phenotype. As more sequence information involving early-replicating isolates becomes available, and additional genotypic signatures are identified, it will be interesting to determine how they influence α_4_β_7_-reactivity. The number of gp120s analyzed here is small; therefore, it will be important to analyze the α_4_β_7_-reactivity of additional early-transmitting gp120s. Definition of the distinguishing features of early-transmitting gp120s is clearly relevant to subunit-based vaccine design.

The influence that PNGs can exert on gp120 structure and immunogenicity has been well studied[66]. The transmission-linked glycans in V1/V2 can, when they are present on a gp120, promote conformational masking. This term is used to describe the capacity of V1/V2 to increase the resistance of HIV-1 isolates to neutralizing antibodies with specificities throughout gp120[41], [42], [43], [44]. This effect has also been referred to as “entropic masking” or “flickering”, both of which describe the property of envelope spikes to rapidly transition between alternate conformations. Rapidly alternating conformations has the net effect of reducing the apparent affinity of neutralizing gp120 antibodies. We find that PNGs, which have been shown to promote conformational masking [44], [46], are among those that can influence α_4_β_7_-reactivity. The manner in which high affinity for α_4_β_7_ is related to conformational masking is an important question that requires further investigation. In any case, our demonstration that the early-transmitting gp120s we analyzed (205F.ENV1.1 0 month founder, CAP88.1m and QA203M1) rely on avidity effects to engage sCD4 suggests that there are structural properties that distinguish these gp120s from many commonly studied gp120s.

Finally, apart from the early-transmitting gp120s, strong α_4_β_7_-reactivity was infrequent among the panel of gp120s that we analyzed. This raises the question as to how frequently viruses bearing this phenotype appear in the viral quasispecies throughout the course of HIV disease. It may be that they appear spontaneously. This seems to be the case in patient 205F in which only the early-transmitting 0 month founder exhibited strong α_4_β_7_-reactivity. It is also possible that viruses bearing this phenotype are compartmentalized in mucosal tissues such that sampling virus from peripheral blood may lead to an underestimation of their frequency. In this regard, it is important to understand how frequently these viruses appear in the donor genital fluids from which virus is transferred to a recipient during sexual transmission.

In conclusion, the transmission-linked PNGΔs that characterize some early-transmitting HIV-1 gp120s mediate a phenotype that includes increased α_4_β_7_-reactivity, suggesting that virions that react strongly with α_4_β_7_ may possess increased transmission-fitness. Further studies will be required to establish a direct link between α_4_β_7_-reactivity and increased transmission across mucosal surfaces. Our observations also suggest that α_4_β_7_^+^/CD4^+^ T-cells are an important target population in the process of transmission. The gp120s we analyzed that exhibited strong α_4_β_7_-reactivity interacted with sCD4 in a way that distinguishes them from many gp120s, and suggests that early-transmitting gp120s bear structural features that might be exploited in the context of a subunit vaccine. Additional genotypic, phenotypic and structural analyses of early-transmitting viral envelopes are essential.

## Materials and Methods

### Ethics statement

PBMCs were collected from healthy donors through a NIH Department of Transfusion Medicine protocol that was approved by the Institutional Review Board of the National Institute of Allergy and Infectious Diseases, National Institutes of Health. Informed consent was written and was provided by study participants and/or their legal guardians.

### Cells and reagents

Freshly isolated PBMCs were obtained from healthy donors and separated by Ficoll-Hypaque. Purified CD4^+^ T cells were obtained by negative selection using magnetic beads (StemCell Technologies). Cultured CD4^+^ T cells were activated with OKT3, IL2 (20 IU/ml) and retinoic acid (10 nM) unless otherwise specified. RA was obtained from Sigma Chemical and discarded 1-month after reconstitution. CHO lec1 cells and HEK293T cells were obtained from ATCC. CHO-S and 293F cells were obtained from Invitrogen. Integrin antibodies were purchased from BD Biosciences, Beckman Coulter and R&D. Leu3A (SK3) and CD45RO were purchased from BD Biosciences.

### Recombinant envelope proteins

The genbank accession numbers of all gp120s used in this study are listed below. The QA203M1 and QA203M41 chimeric gp120s were constructed by inserting the V1/V2 sequences of each of these gp120s into a Q23 backbone. QA203M1: VTLECSNVNVTNNVTNDMGEEIKNCSFNMTTELRDKKQKTYSLFYKLDVVPFNNRSQYRLIN.QA203M41: VTLECSNVNVTNNVNVTNNVNVTNNVTNDMTGEIKNCSFNMTTELRDKKQKVYSLFYKLDVVPVNNNSSQYRLIN. All gp120 coding sequences were synthesized and codon-optimized for expression in mammalian cells (DNA2.0). All proteins were produced and purified in an identical manner unless noted otherwise. The mature coding sequences of each envelope protein, from +1 to the gp120-gp41 junction were inserted into a mammalian expression vector downstream of a synthetic leader sequence. Vectors were transiently transfected into either 293F or CHO-S cells (Invitrogen) using FreeStyle MAX Reagent (Invitrogen) per the manufacturers instructions. gp120s expressed in CHOlec1 cells were transfected by CaPO_4_ and stable cell lines were selected in media containing 1 mg/ml G418. Clonal cell lines were established and subsequently seeded into hollow-fiber cartridges (30 kD MW cutoff) (Fibercell systems, Frederick MD). Protein containing supernatants were harvested daily from the extra-capillary space. Protein containing supernatants were harvested and passed over a column of *galanthus nivalis* lectin sepharose (Vector Labs) which was diluted 1∶5 with unliganded sepharose 4B to minimize avid binding. gp120 was eluted with 20 mM Glycine-HCl, pH 2.5, 150 mM NaCl, 500 mM α-methyl-manno pyranoside (Sigma), in 5 mL fractions directly into 1 mL M Tris-HCL, pH 8.0. Low pH elution was found to be necessary with certain gp120s which bound too tightly to the lectin to be efficiently eluted using α-methyl-manno pyranoside. Peak fractions were pooled, concentrated with a stirred cell concentrator (Millipore) and dialyzed exhaustively against HEPES, pH 7.4, 150 mM NaCl. Proteins were quantitated by UV adsorption at O.D. λ_280_ (extinction coefficient 1.1) and values were confirmed by a bicinchoninic acid protein assay (Pierce).

### Recombinant gp120 protein labeling

Purified recombinant gp120s were biotinylated using amine-coupling chemistry. Proteins were reacted with a 100-fold molar excess of EZ-Link NHS-Biotin (Pierce) for 30 min, and reactions were quenched by rapid buffer-exchange into HBS. Biotin incorporation was determined by reacting gp120s with 4′-hydroxyazobenzene-2-carboxylic acid-avidin conjugates (HABA) per the manufacturers instructions (Pierce). Protein preparations exhibiting a 1.0–1.2 mol/mole, biotin/gp120 incorporation were used in comparative semi-quantitative flow-cytometric binding assays.

### Flow cytometry binding assays

CD4^+^ T cells were cultured in RA for at least 6 days prior to use, and were stained with fluoresceinated anti β_7_ mAb FIB27 (ATCC) to insure RA-mediated upregulation of α_4_β_7_. Receptor quantitation using anti-mouse IgG coated microbead analysis of CD4 and β_7_ expression levels of these cells from multiple donors indicates that levels of β_7_ expressed on the surface of these cells typically lower and less uniform than CD4 expression levels (data not shown). The entire staining procedure, including wash steps was carried out in a 10 mM HEPES, 150 mM NaCl (HBS Buffer) buffer containing 100 µM CaCl_2_ and 1 mM MnCl_2_. Cells were pre-blocked with normal mouse IgG and human IgG (5 µg each per 10^6^ cells. 3×10^5^ cells were stained in a volume of 50 µl on ice. Where indicated CD4-gp120 interactions were masked by preincubating cells for 15′ with 5 µg Leu3A (SK3) (Becton Dickinson). Where indicated α_4_β_7_-gp120 interactions were masked with 5 µg unlabeled α_4_ mAb HP2/1 (Beckman Coulter). Masking antibodies were not washed away prior to gp120 staining. Biotin gp120 was added for 25′ on ice, after which cells were washed twice with staining buffer. In some assays CD45RO FITC and CCR5 APC were included, Neutravidin PE (Pierce) was then added, and incubation proceeded for an additional 30′ on ice. Cells were washed three times in staining buffer and then fixed in a 1% paraformaldehyde solution. Data were acquired using a BD FACSCalibur and mean fluorescence intensity measurements were obtained from the CD45RO^+^/CCR5^+^ gate.

### Surface plasmon resonance analysis

Analysis was performed on a Biacore 3000 instrument (GE Life Sciences) using CM5 sensor chips. The data were evaluated using BIAevaluation 4.1 software (GE Life Sciences). The chip surface was activated by injecting 35 µl of a 1/1 mixture of 0.05 M *N*-hydroxysuccinimide and 0.2 M *N*-ethyl-*N*-(dimethylaminopropyl)carbodiimide at 5 µl/min. Purified gp120 (5 µg/ml in 10 mM NaOAc (pH 5)) was immobilized to a density of approximately 750 resonance units (RU) and blocked with 35 µl of 1 M Tris-HCl (pH 8.0). Human IgG was immobilized to one flow cell and used as a background control. Running buffer was HBS (pH 7.4), 0.005% Tween p20. To evaluate Env: CD4 interactions, increasing concentrations of sCD4 (D1D2) (6.25–400 nM) were sequentially injected over surface-bound gp120 at a flow rate of 25 µl/min. After a 2 min injection of sCD4, the surface was washed for 2 min in running buffer to follow the dissociation of sCD4 from gp120. The surfaces were regenerated by injection of 4.5 M MgCl_2_ at a flow rate of 50 µl/min.

### Genbank accession numbers of gp120s

The following gp120s were employed in these studies: 93MW959 (GenBank accession # U08453, R5-tropic), 92TH14-12 (GenBank accession #U08801, R5-tropic), 93Ug037 (GenBank accession # U51190, R5-tropic), AN1 gp120 [35] (sequence available at http://ubik.mullins.microbiol.washington.edu/HIV/Doria-Rose2005/, R5-tropic), 92Ug21-9 (GenBank accession # U08804, X4-tropic).), ADA (GenBank accession #AF004394, R5-tropic), YU-2 (GenBank accession #M93258, R5-tropic), NL4-3 (GenBank accession # AF003887, X4-tropic), 92Ug21-9 (GenBank accession #AY669753, X4-tropic), and SF162 (GenBank accession # AY669736, R5-tropic). Z205F.ENV1.1 0Mfounder (GenBank accession #GQ485415, R5-tropic) Z205FENV6.3 0Mescape GenBank accession #GQ485419, R5-tropic), Z205FENV2.3 8Mescape (GenBank accession # GQ485425, R5-tropic), Z205FENV5.1 38Mescape GenBank accession #GQ485445, R5-tropic), Z205FENV4.1 38Mescape GenBank accession # GQ485447, R5-tropic) QA203M1 (GenBank accession #DQ136332, R5-tropic) QA203M41 (GenBank accession #DQ136341, R5-tropic), Q23 (GenBank accession # AF004885, R5-tropic).

## Supporting Information

Figure S1 Flow cytometry based α_4_β_7_ steady-state binding assay. A) The α_4_β_7_-binding assay employed highly activated purified CD4^+^ T cells cultured for 6–9 days in retinoic acid. α_4_β_7_ expression was monitored by staining cells with CD45RO and the β_7_ mAb FIB27. B) Gating on the CD45RO/CCR5 population was carried out in order to analyze gp120 binding to α_4_β_7_ high CD4^+^ T cells. C) α_4_β_7_-reactivity was reported as the mean fluorescence intensity of biotinylated gp120s binding to the CCR5^+^/CD45RO^+^ cell subset. Binding assays were carried out in the presence of an unlabeled CD4 mAb (Leu3A/SK3) in order to block gp120 binding to CD4. Where specified an unlabeled α_4_ mAb (HP2/1) was used to mask α_4_β_7_. Specificity was demonstrated by masking with both mAbs. D) In most assays gp120 reactivities, in the presence of excess unlabeled Leu3A, were measured over a range of concentrations, and steady-state reactivity was determined following a 30-minute incubation at 4°C. HP2/1 was included along with Leu3A as a specificity control, where indicated.
(2.46 MB TIF)

Figure S2 Comparison of the α_4_β_7_-reactivity of CHO-S vs. 293F produced gp120. Flow-cytometry based measurement of the α_4_β_7_-reactivity of CAP881m.C12 gp120 expressed in either CHO-S cells or 293F cells. Reactivity to both CD4 and α_4_β_7_ was measured by differentially masking each receptor with unlabeled mAbs. Values reported reflect mean fluorescence intensity (MFI). These results are representative of three independent experiments.
(0.13 MB TIF)

Figure S3 The effect of Endoglycosydase H treatment of AN1 gp120 on α_4_β_7_-reactivity. AN1 gp120 (subtype B) was either mock- or endoglycosidase H-digested for 50 and 250 minutes, and α_4_β_7_-reactivity was determined by binding to α_4_β_7_ high CD4^+^ T cells as described in Figure S1. Values reported reflect mean fluorescence intensity (MFI). These results are representative of three independent experiments.
(0.13 MB TIF)

Figure S4 Amino acid sequences of early-transmitting gp120s and neutralization escape variants. The V1/V2 sequences of patient 205F gp120s analyzed in this study, and the V1/V2 and C3/V4 sequences of the patient CAP88 gp120s analyzed in this study deposited in GENBANK by the referenced investigators. Amino acid substitutions in the CAP88 12 month isolate that contribute to Nab escape are highlighted in red.
(0.57 MB TIF)

Figure S5 Surface plasmon resonance analysis of sCD4 (D1D2) binding to immobilized gp120s. Sensorgrams depicting the binding kinetics of increasing concentrations of monomeric sCD4 D1D2 reacting with a panel of immobilized gp120s. Each ligand/analyte pair is listed and overall affinity is reported as both K_D_ and K_A_. On-rates (k_a_) and off-rates (k_d_) are also listed.
(1.44 MB TIF)
