# Supplementary figures and images for: The Genotype of Early-Transmitting HIV gp120s Promotes α4β7 –Reactivity, Revealing α4β7 +/CD4+ T cells As Key Targets in Mucosal Transmission
Source: PLoS Pathog. 2011 Feb 24;7(2):e1001301. doi: 10.1371/journal.ppat.1001301 (PMC3044691; doi:10.1371/journal.ppat.1001301)

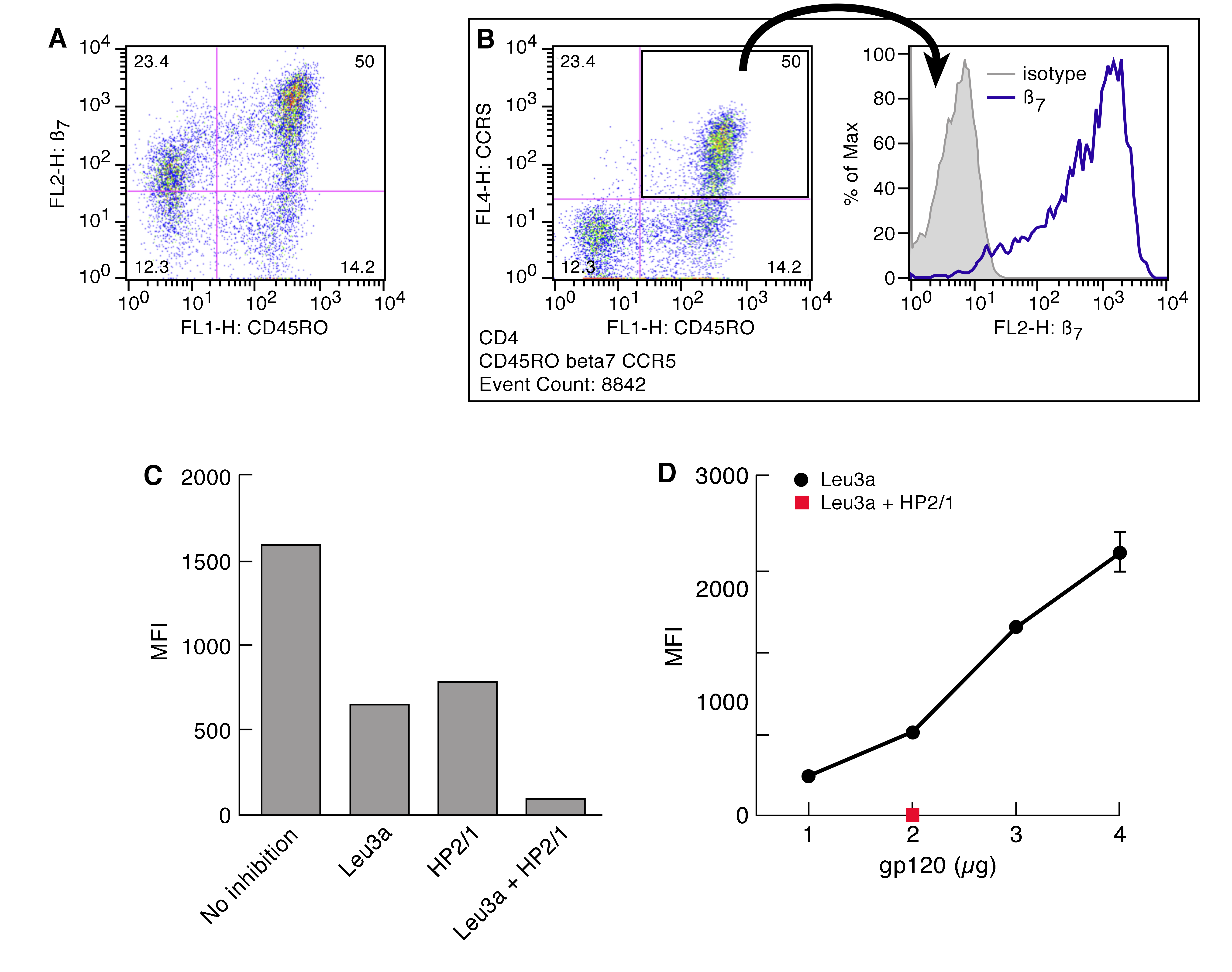

Supplement: Figure S1 — Flow cytometry based α4β7 steady-state binding assay. A) The α4β7-binding assay employed highly activated purified CD4+ T cells cultured for 6–9 days in retinoic acid. α4β7 expression was monitored by staining cells with CD45RO and the β7 mAb FIB27. B) Gating on the CD45RO/CCR5 population was carried out in order to analyze gp120 binding to α4β7 high CD4+ T cells. C) α4β7-reactivity was reported as the mean fluorescence intensity of biotinylated gp120s binding to the CCR5+/CD45RO+ cell subset. Binding assays were carried out in the presence of an unlabeled CD4 mAb (Leu3A/SK3) in order to block gp120 binding to CD4. Where specified an unlabeled α4 mAb (HP2/1) was used to mask α4β7. Specificity was demonstrated by masking with both mAbs. D) In most assays gp120 reactivities, in the presence of excess unlabeled Leu3A, were measured over a range of concentrations, and steady-state reactivity was determined following a 30-minute incubation at 4°C. HP2/1 was included along with Leu3A as a specificity control, where indicated. (2.46 MB TIF) [file ppat.1001301.s001.tif]

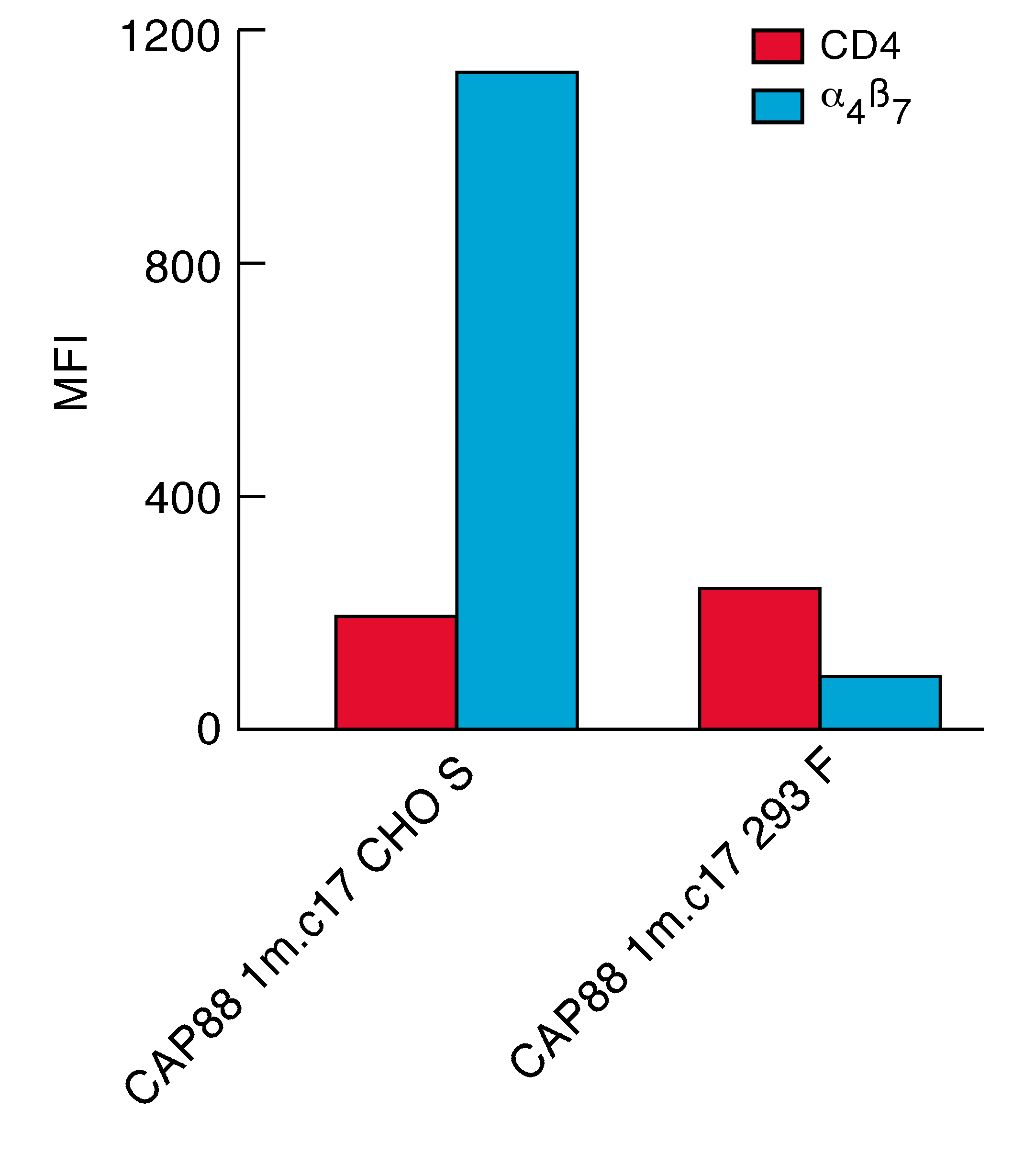

Supplement: Figure S2 — Comparison of the α4β7-reactivity of CHO-S vs. 293F produced gp120. Flow-cytometry based measurement of the α4β7-reactivity of CAP881m.C12 gp120 expressed in either CHO-S cells or 293F cells. Reactivity to both CD4 and α4β7 was measured by differentially masking each receptor with unlabeled mAbs. Values reported reflect mean fluorescence intensity (MFI). These results are representative of three independent experiments. (0.13 MB TIF) [file ppat.1001301.s002.tif]

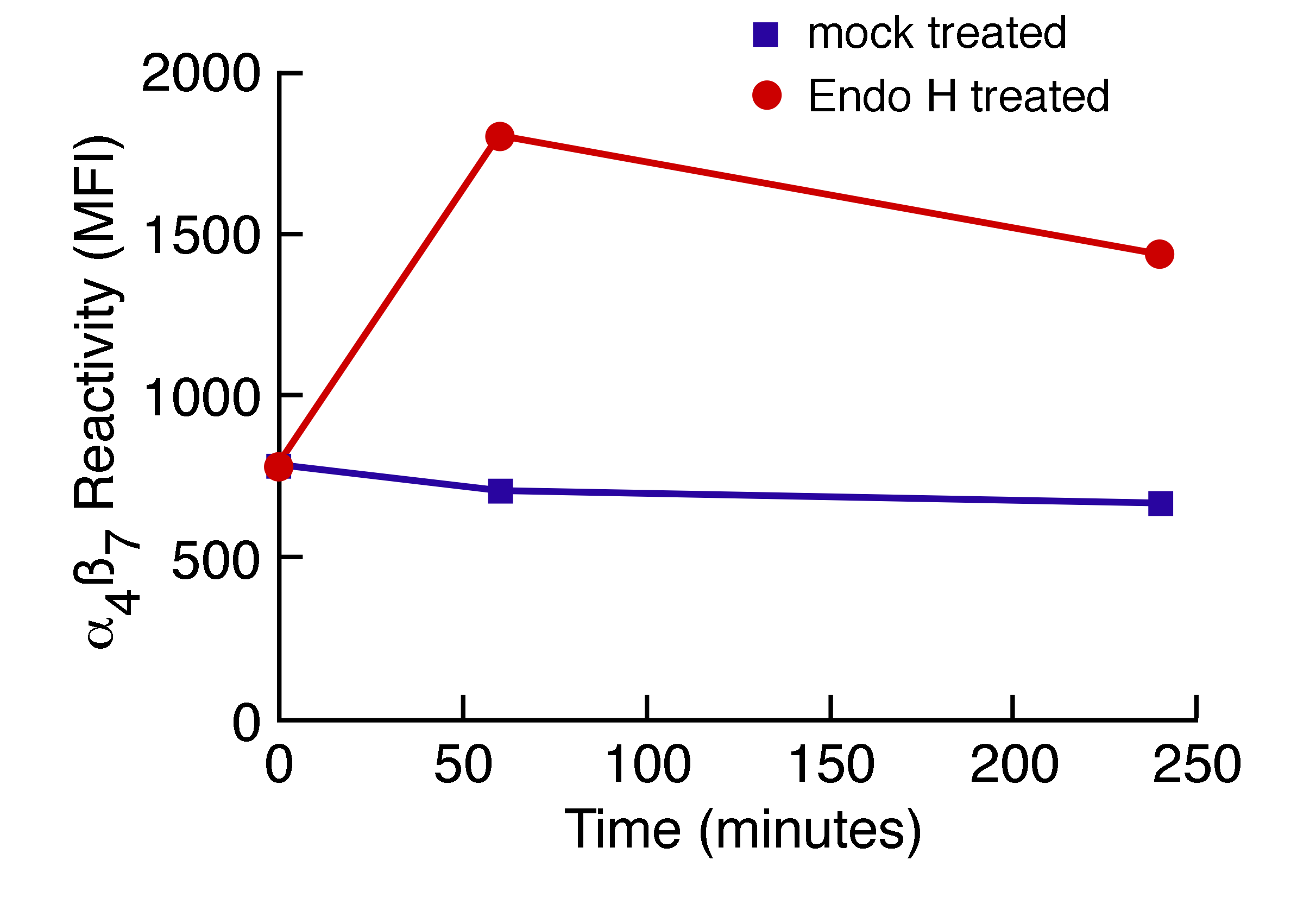

Supplement: Figure S3 — The effect of Endoglycosydase H treatment of AN1 gp120 on α4β7-reactivity. AN1 gp120 (subtype B) was either mock- or endoglycosidase H-digested for 50 and 250 minutes, and α4β7-reactivity was determined by binding to α4β7 high CD4+ T cells as described in Figure S1. Values reported reflect mean fluorescence intensity (MFI). These results are representative of three independent experiments. (0.13 MB TIF) [file ppat.1001301.s003.tif]

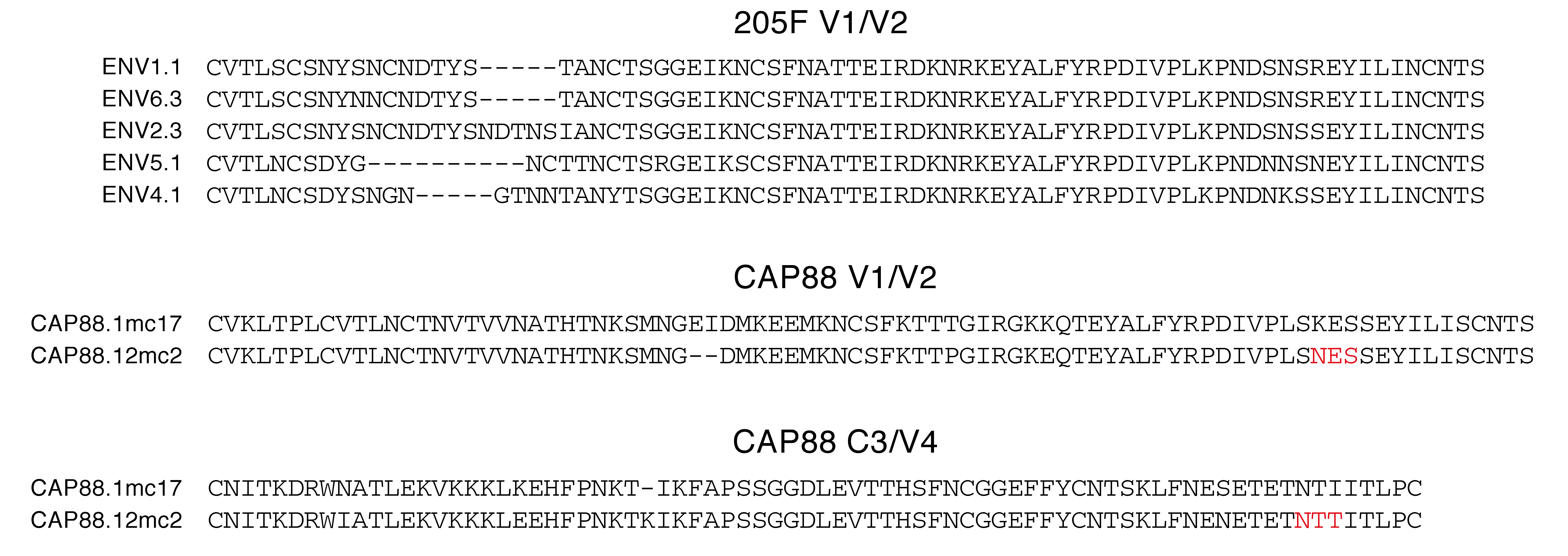

Supplement: Figure S4 — Amino acid sequences of early-transmitting gp120s and neutralization escape variants. The V1/V2 sequences of patient 205F gp120s analyzed in this study, and the V1/V2 and C3/V4 sequences of the patient CAP88 gp120s analyzed in this study deposited in GENBANK by the referenced investigators. Amino acid substitutions in the CAP88 12 month isolate that contribute to Nab escape are highlighted in red. (0.57 MB TIF) [file ppat.1001301.s004.tif]

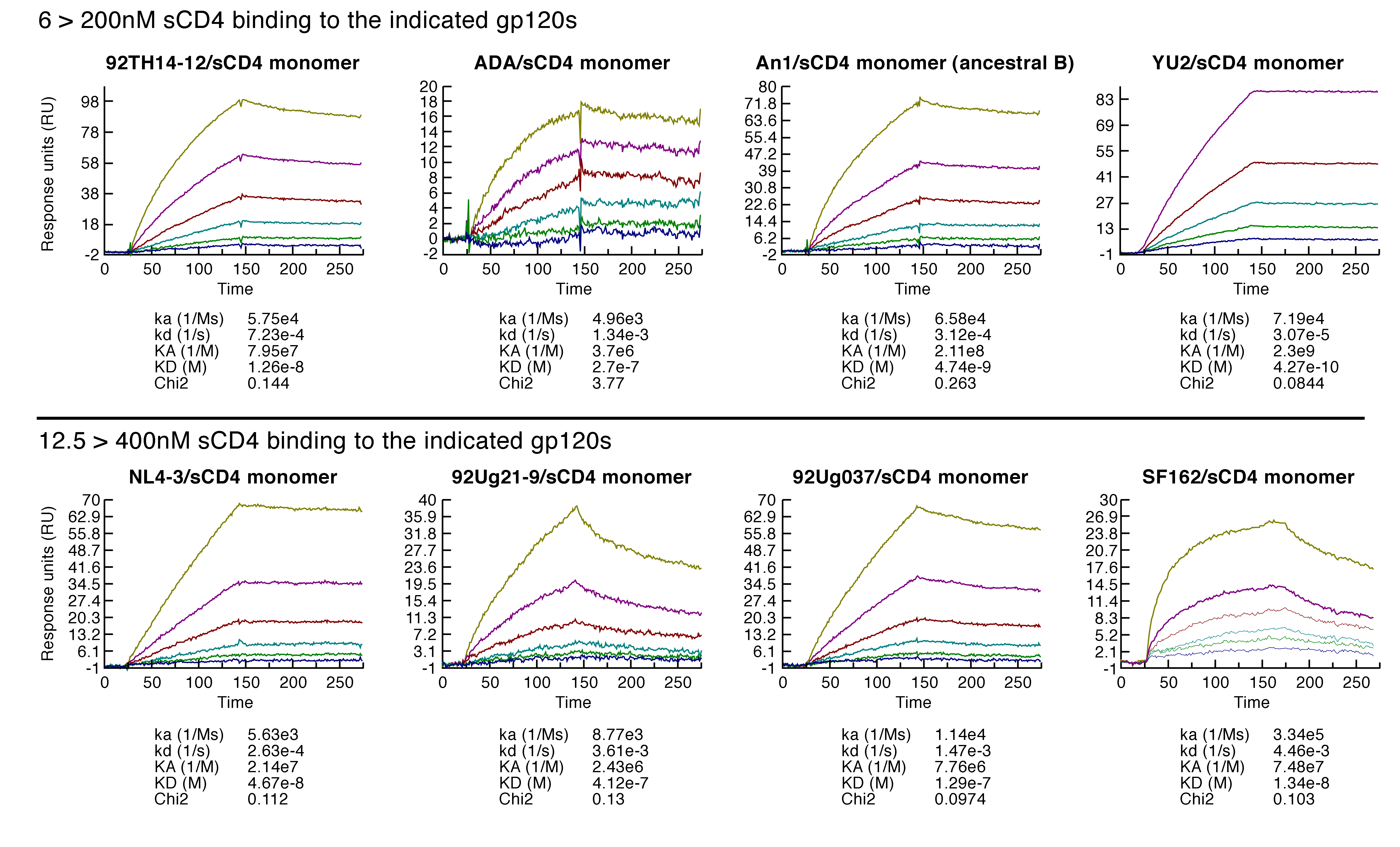

Supplement: Figure S5 — Surface plasmon resonance analysis of sCD4 (D1D2) binding to immobilized gp120s. Sensorgrams depicting the binding kinetics of increasing concentrations of monomeric sCD4 D1D2 reacting with a panel of immobilized gp120s. Each ligand/analyte pair is listed and overall affinity is reported as both KD and KA. On-rates (ka) and off-rates (kd) are also listed. (1.44 MB TIF) [file ppat.1001301.s005.tif]
